# One-Pot Hydrothermal Synthesis of MoS_2_/Zn_0.5_Cd_0.5_S Heterojunction for Enhanced Photocatalytic H_2_ Production

**DOI:** 10.3389/fchem.2020.00779

**Published:** 2020-09-03

**Authors:** Xinru Li, Fei Xue, Naixu Li, Xukai Wei, Hui Liu, Jianchen Zhou, Bin Lyu, Maochang Liu

**Affiliations:** ^1^School of Material Science and Engineering, Shaanxi University of Science and Technology, Xi'an, China; ^2^College of Petroleum Engineering, Xi'an Shiyou University, Xi'an, China; ^3^International Research Center for Renewable Energy, State Key Laboratory of Multiphase Flow in Power Engineering, Xi'an Jiaotong University, Xi'an, China; ^4^School of Chemistry and Chemical Engineering, Southeast University, Nanjing, China; ^5^College of Bioresources Chemical and Materials Engineering, Shaanxi University of Science and Technology, Xi'an, China; ^6^National Educational Reform Experimental Demonstration Center, Xi'an, China

**Keywords:** photocatalysis, hydrogen production, sulfide, heterojunction, cocatalyst

## Abstract

A series of molybdenum disulfide (MoS_2_)/Zn_0._5Cd_0.5_S heterojunctions have been prepared via a mild one-pot hydrothermal method based on the optimization of composition content of primary photocatalyst. The photocatalysts demonstrated significantly improved visible light–driven photocatalytic activity toward H_2_ evolution from water without using any noble metal cocatalyst. Among the as-prepared composites, 0.2% MoS_2_/Zn_0.5_Cd_0.5_S shows the best performance. The highest H_2_ evolution rate reaches 21 mmol · g^−1^ · h^−1^, which is four times higher than that of pure Zn_0.5_Cd_0.5_S. The apparent quantum efficiency is about 46.3% at 425 nm. The superiority is attributed to the tight connection between MoS_2_ and Zn_0.5_Cd_0.5_S by this facile one-step hydrothermal synthesis. As a result, the formation of the heterostructure introduces built-in electric field at the interface that facilitates vectorial charge transfer. More specifically, photogenerated electrons transfer to MoS_2_ to conduct proton reduction, where the holes are retained on the surface of Zn_0.5_Cd_0.5_S to react with the sacrificial reagents. Moreover, the composite presents improved stability without notable activity decay after several cycled tests.

## Introduction

Derived from the high heat of combustion and carbon-neutral energy cycle, hydrogen energy has been envisaged as an appealing substitute for excessively depleted fossil resources (Kim et al., 2014). Since Fujishima and Honda (1972) as the pioneers discovered water photolysis on TiO_2_ electrode in 1972, solar H_2_ production on semiconductor-based photocatalysts has been widely accepted as an ideal strategy for addressing the imminent energy crisis and incremental pollution issues (Guo et al., 2018; Liu et al., 2018; Yang Y. et al., 2018; Jiao et al., 2020). The achievement of this technology lies in rational design and development of efficient and cost-effective photocatalysts with high quantum efficiency (Yan et al., 2009, 2011; Lin et al., 2017; Huckaba et al., 2018; Singh et al., 2018). Particularly, the visible light accounted for 43% of the whole solar spectrum (Guo et al., 2016). Therefore, in the past few decades, much effort has been attempted to synthesize visible light–driven photocatalysts (Tan et al., 2016; Liu et al., 2017, 2018; Feng et al., 2018; Hafeez et al., 2018; Jin et al., 2018). Among the various photocatalysts, metal sulfides have been confirmed to be promising assignable to their superior photocatalytic performance (Tsuji et al., 2004; Iwashina et al., 2015; Di et al., 2016; Zhai et al., 2018). Specially, CdS is deemed as one of the most appropriate binary chalcogenide photocatalyst owing to the narrow band gap (~2.4 eV) for visible light response and the more negative conduction band position than reduction potential of H^+^/H_2_ (0 V vs. NHE, pH = 0) (Chao et al., 2017; Zhou et al., 2017; Yuan et al., 2018). Despite these inherent advantages of CdS photocatalyst, photocorrosion and toxicity should not be ignored. Motivated by well-matched coordination mode between CdS and ZnS, embedding Zn^2+^ into crystal cell of CdS to fabricated solid solution (Zn_*x*_Cd_1−x_S) has been found to increase the stability and photoactivity simultaneously (Liu et al., 2011, 2013; Yang M. et al., 2018).

To further boost the photoactivity of Zn_*x*_Cd_1−x_S photocatalysts, integrating suitable cocatalysts to trap charges carriers and serve as delicate surface reactive sites would be a productive approach. However, as the most common cocatalyst, the application of noble metals (e.g., Pt and Pd) can be confined only to bench scale because of their high cost and scarcity (Gao et al., 2019; Li et al., 2020a,b). Therefore, the exploration and development of alternative noble metal–free but active cocatalysts are highly desired. Up to now, plenty of earth-abundant transition metal compounds are developed as efficient and non-precious cocatalysts (Zong et al., 2008; Akple et al., 2015; He et al., 2017; Zhang et al., 2017, 2018; Xu et al., 2018). Among them, molybdenum disulfide (MoS_2_) with layered structures has attracted wide attention because of optimized surface binding free energy of H atoms, large layered structure determined surface area, and excellent structural adjustability (Frame et al., 2010; Li et al., 2013; Qi et al., 2013; Chung et al., 2014; He et al., 2016; Zeng et al., 2017). Furthermore, some other works disclosed the superior cocatalytic function of MoS_2_ over TiO_2_, g-C_3_N_4_, and CdS, indicating its general applicability (Zhou et al., 2012; Parayil et al., 2013; Chai et al., 2018; Zhang et al., 2018). It is highly noted that the assembly of MoS_2_ and primary photocatalysts is usually achieved by the time-consuming two-step method, bringing about a weak interfacial interaction for charge transfer and inferior stability (Zong et al., 2008; Frame et al., 2010; Zhou et al., 2012; He et al., 2016). In contrast, one-pot hydrothermal approach has been certified to be feasible to fabricate tight connected interface between photocatalyst and cocatalyst. It can be thus expected that MoS_2_ could integrate on the Zn_*x*_Cd_1−x_S surface with enhanced physicochemical property.

Herein, we reported a simple and low-cost one-step hydrothermal method for coating MoS_2_ onto the surface of Zn_*x*_Cd_1−x_S directly. The series of photocatalysts with different weight ratio between MoS_2_ and Zn_*x*_Cd_1−x_S exhibited superior photocatalytic performance toward H_2_ evolution within Na_2_S/Na_2_SO_3_ aqueous solution. When the capacity of MoS_2_ is 0.2%, the highest activity was approached, with an H_2_ production rate of 21 mmol · h^−1^ · g^−1^. Moreover, the durability was further improved by the incorporation of MoS_2_. The improvement dominantly depends on the smooth transfer of electrons through the intimate interface induced by the one-pot hydrothermal route and promoted surface redox reaction at the numerously increased active sites. It is believed that this work could inspire the application of other noble metal–free cocatalyst decorated photocatalyst with highly efficient photocatalytic activity.

## Experimental Section

### Chemicals and Materials

Cadmium acetate [Cd(CH_3_COO)_2_ · 2H_2_O], zinc acetate [Zn(CH_3_COO)_2_ · 2H_2_O], thioacetamide (TAA, C_2_H_5_NS), sodium molybdate (Na_2_MoO_4_ · 2H_2_O), sodium sulfide (Na_2_S·9H_2_O), sodium sulfite (Na_2_SO_3_), and ethanol (CH_3_CH_2_OH) were used as received. The water used in all syntheses was deionized (DI) water with a resistivity of 18.2 MΩ · cm.

### One-Pot Hydrothermal Synthesis of Zn*_*x*_*Cd_1-*x*_S Solid Solution

TAA 50 mmol was first dissolved into 35 mL ethanol. Then the ethanol solution was mixed with 35 mL aqueous solution containing certain amount of Cd(CH_3_COO)_2_ · 2H_2_O and Zn(CH_3_COO)_2_ · 2H_2_O (the total mole number of Cd and Zn precursor is 20 mmol). After stirring for 20 min at 800 revolutions/min (rpm), the suspension was transferred to the 100 mL Teflon-lined stainless steel autoclave, followed by heat treatment at 200°C for 48 h. Based on cooling down naturally, the obtained product was centrifuged and washed with ethanol and deionized water for several times. A yellow powder is prepared by drying the product under vacuum at 80°C for 5 h. Finally, prepared samples were labeled as Zn_*x*_Cd_1−x_S, where *x* equals 0, 0.1, 0.3, 0.5, 0.7, 0.9, and 1, respectively, indicating the stoichiometric ratio between Zn and S.

### One-Pot Hydrothermal Synthesis of MoS_2_/Zn_0.5_Cd_0.5_S Nanocomposites

Ethanol 35 mL and TAA 50 mmol were dissolved into a 35 mL aqueous solution containing 10 mmol Cd(CH_3_COO)_2_ · 2H_2_O and 10 mmol Zn(CH_3_COO)_2_ · 2H_2_O. Then 5 mL aqueous solution containing a certain amount Na_2_MoO_4_ · 2H_2_O (0; 0.002; 0.004; 0.006 mmol) was added to it with continuous stirring at 800 rpm. After that, the mixture was transferred to a 100 mL Teflon-lined autoclave and heated at 200°C for 48 h. After cooling down to room temperature, the resultant product was obtained by centrifugation and washed with deionized water and ethanol for several times thoroughly. Then it was dried at 80°C in a vacuum oven. According to the difference of concentrations of MoS_2_, the final MoS_2_/Zn_0.5_Cd_0.5_S was designed as Zn_0.5_Cd_0.5_S, 0.1% MoS_2_/Zn_0.5_Cd_0.5_S, 0.2% MoS_2_/Zn_0.5_Cd_0.5_S, and 0.3% MoS_2_/Zn_0.5_Cd_0.5_S, respectively.

### Photocatalytic Reactions

The photocatalytic performance of various photocatalysts from the same batch was estimated one after another. Photocatalytic reactions of hydrogen production from water were conducted in a gas-closed system with a side irradiation Pyrex cell (using PLS-SXE300/300UV Xe lamp irradiation) at 35°C. The photocatalyst powder was dispersed by a magnetic stirrer in an aqueous solution (180 mL) containing Na_2_S (0.35 M) and Na_2_SO_3_ (0.25 M) as electron donors. After being evacuated by N_2_ gas with a heavy flow for over 20 min, the photocatalysts were irradiated with visible light (λ ≥430 nm) through a cutoff filter from the Xe lamp. The intensity and number of photons of the light source were measured by a fiber-optic spectrometer. The amount of H_2_ gas was determined using online gas chromatography (NaX zeolite column, TCD detector, N_2_ carrier) or drainage at every hour interval. Blank experiments showed that no H_2_ was produced. Apparent quantum yield (AQY) defined by Equation (1) was measured using a 425-nm band-pass filter and an irradiate meter.

AQY = the number of reacted electrons/ the number of incident photons = the number of evolved H_2_ molecules × 2/the number of incident photons                     (1)

### (Photo)electrochemical Measurements

A CHI760d electrochemical workstation (Chenhua Instruments Co., Shanghai, China) was applied to record transient photocurrent response, electrochemical impedance spectra (EIS), and Mott–Schottky (MS) plot. In a typical (photo)electrochemical test, a standard three-electrode system with the sample as working electrode, Ag/AgCl (in saturated KCl) as reference electrode, Pt wire as counter electrode, and sodium sulfate aqueous solution (0.5 M) as the electrolyte was employed. An Xe lamp with a simulated solar light filter (AM 1.5) was adopted as the light source for transient photocurrent response tests. For the preparation of working electrodes, 1 mg of as-synthesized samples and 20 μL of 5% Nafion (DuPont) were uniformly dispersed in 1 mL of water–ethanol mixture solution. After ultrasonication treatment to the mixture for 1 h, 0.5 mL of homogeneous suspension was dropped onto a 2 × 3-cm fluorine-doped tin oxide glass plate. When the samples were naturally dried under ambient temperature, the working electrodes could be obtained.

### Characterizations

X-ray diffraction (XRD) patterns of prepared photocatalysts were confirmed by an X'Pert PRO diffractometer using Cu K_α_ (λ = 0.1538 nm) irradiation with constant instrument parameter. Diffuse reflectance ultraviolet-visible (UV-vis) spectra were measured on a Hitachi U-4100 spectrometer, equipped with a lab-sphere diffuse reflectance accessory. The crystallite morphologic micrographs were observed by JEOL JSM-7800F field emission scanning electron microscopy and FEI Tecnai G2 F30 transmission electron microscope (TEM). The X-ray photoelectron spectroscopy (XPS) measurements were conducted on Axis Ultra, Kratos (UK) multifunctional spectrometer using monochromatic Al K_α_ radiation. The C 1s peak at 284.8 eV of adventitious carbon was used for calibration.

## Results and Discussion

**Figure 1:**
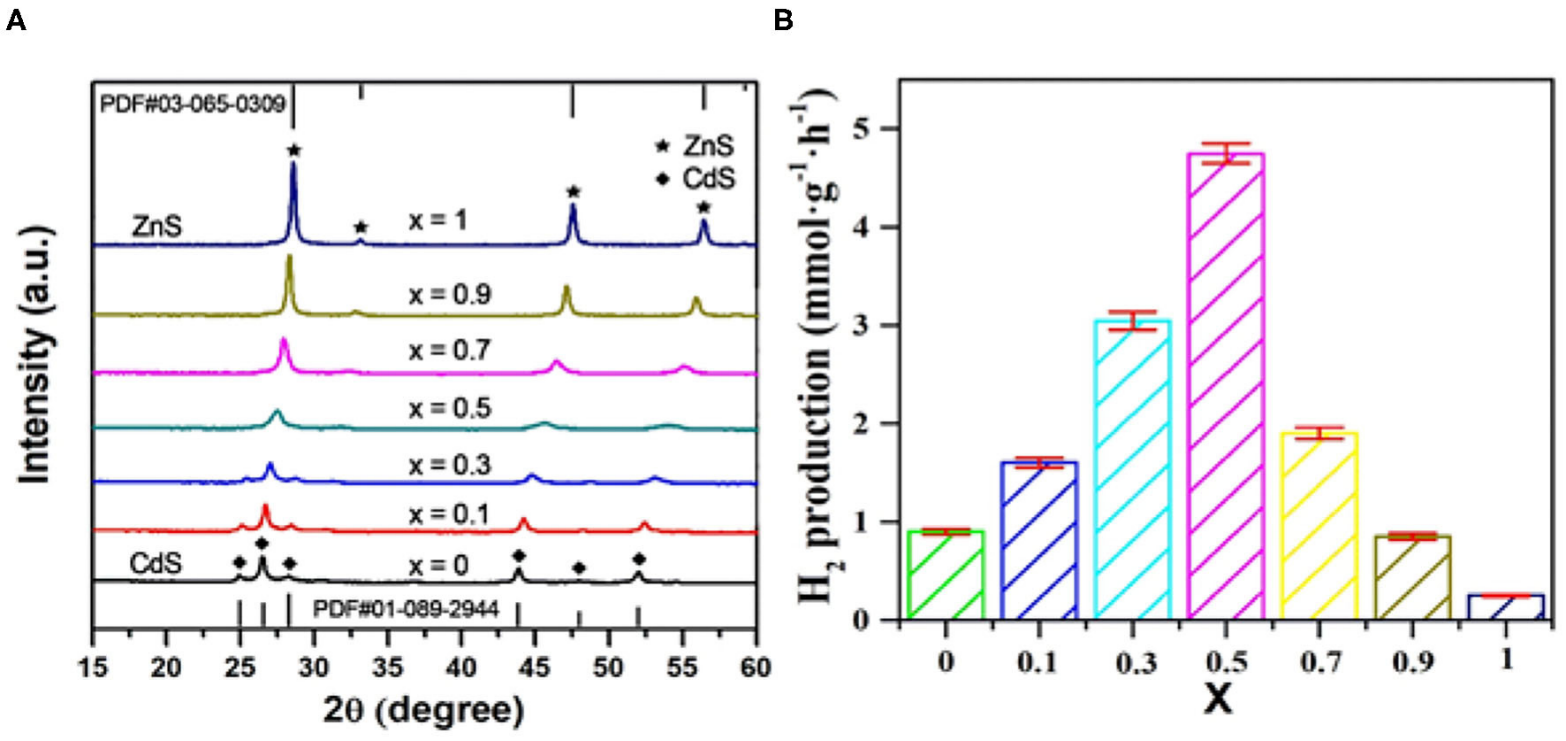
**(A)** XRD patterns and **(B)** photocatalytic performances of as-prepared Zn_*x*_Cd_1−x_S solid solutions.

The prepared composites were first investigated by XRD. As shown in Figure 1A, comparing with the standard XRD patterns (JCPDS 03-065-0309 for zinc-blende ZnS, JCPDS 01-089-2944 for wurtzite-type CdS), the *x*-value–dependent phase transition from wurtzite-type structure (*x* < 0.5) to zinc-blended structure (*x* ≥ 0.5) was definitely observed. Likewise, on the premise of the same phase structure, all the Zn_x_Cd_1−x_S samples manifested a successive and systematical diffraction peak shift to higher angle with the increase of *x*-value, implying the successful formation of solid solution (Yang M. et al., 2018). Photocatalytic tests toward H_2_ evolution over these Zn_*x*_Cd_1−x_S photocatalysts behaved a significant volcano trend along with the change of *x* value (Figure 1B). When the Zn/Cd ratio reached 1:1, Zn_0.5_Cd_0.5_S exhibited the highest activity with an H_2_ evolution rate of 4.5 mmol · g^−1^ · h^−1^. In principle, the continuous incorporation of Zn^2+^ into CdS lattice is expected to sequentially broaden the band gap of the solution. On the one hand, the widen band gap signifies the narrow light absorption range; on the other hand, it also forebodes the enhanced reduction potential of electrons that resulted from the upshifted conduction band position. Therefore, it is of great benefit for improving photocatalytic activity by coordinating the balance between these two contradictions. As a circumstance, Zn_0.5_Cd_0.5_S with the suitable band gap and relatively negative conduction band position showed the superior photoactivity in our case. In subsequent investigation, Zn_0.5_Cd_0.5_S was taken as the primary photocatalyst to integrate with MoS_2_ for construction of composites.

**Figure 2:**
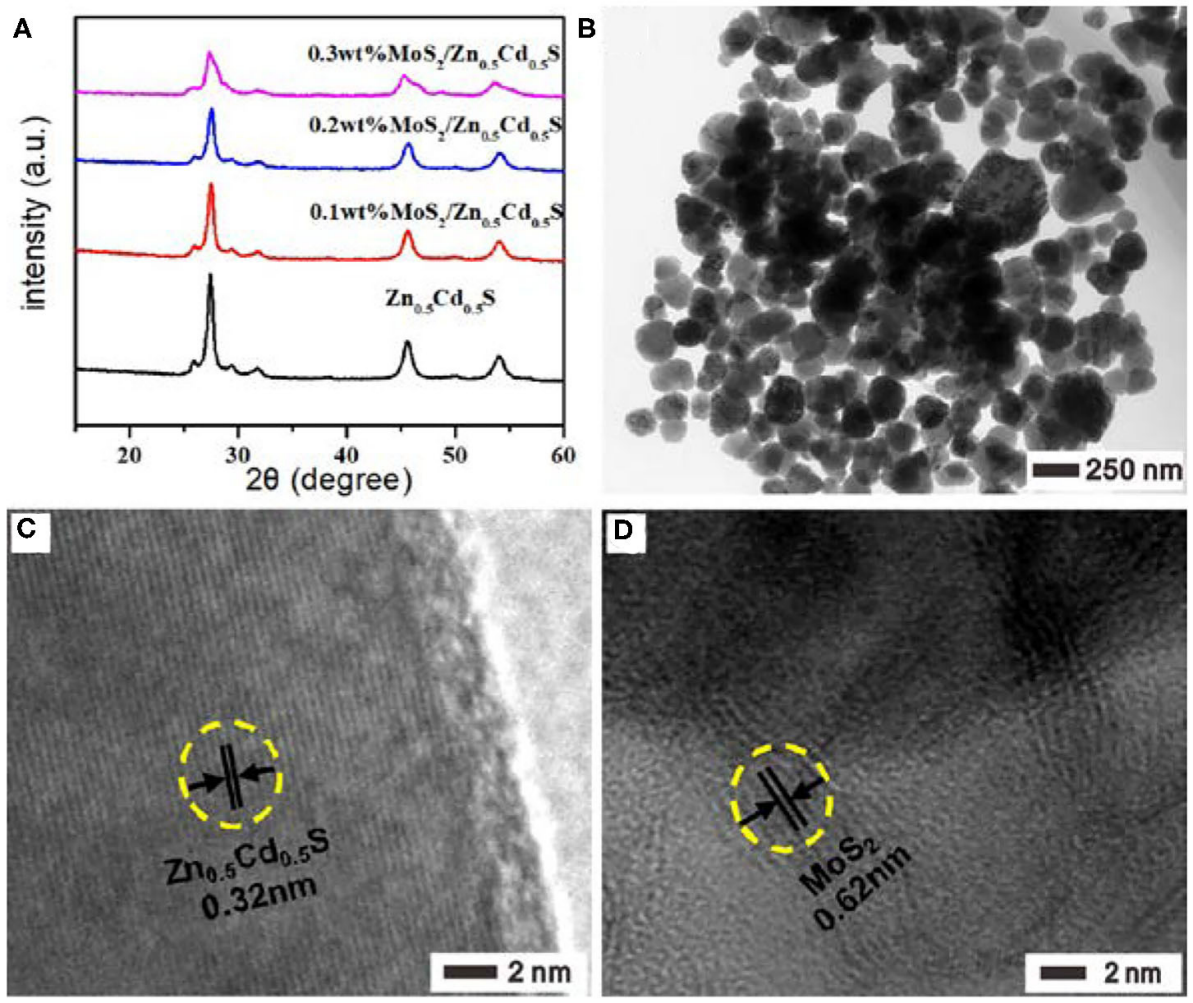
**(A)** XRD patterns of Zn_0.5_Cd_0.5_S, 0.1% MoS_2_/Zn_0.5_Cd_0.5_S, 0.2% MoS_2_/Zn_0.5_Cd_0.5_S, and 0.3% MoS_2_/Zn_0.5_Cd_0.5_S. **(B,C)** TEM images of 0.2% MoS_2_/Zn_0.5_Cd_0.5_S. **(D)** TEM images of 1% MoS_2_/Zn_0.5_Cd_0.5_S.

The MoS_2_ with various loading amounts were then introduced onto the surface of Zn_0.5_Cd_0.5_S via the facile one-pot hydrothermal method. To gain insight into the microstructures, the as-prepared MoS_2_/Zn_0.5_Cd_0.5_S samples were first characterized by XRD. As observed in Figure 2A, all the samples were found with identical diffraction peak positions regardless of the MoS_2_ loading amount, indicating the crystal structure of Zn_0.5_Cd_0.5_S was independent of MoS_2_. Further observation revealed the obviously decreased diffraction intensity at 27.6° with the increase of MoS_2_ introduction, which should be ascribed to the scattering and absorption effect of MoS_2_ to the incident X-ray. This notion also verified the tight connection between these two components indirectly, offering a great opportunity to improve the catalytic properties (Zhang et al., 2019). In contrast, no visible diffraction peaks could be discerned to MoS_2_ because of its small amount exceeding detection limit. The surface morphology and particle sizes, taking 0.2% MoS_2_/Zn_0.5_Cd_0.5_S as a model photocatalysts, were tested by high-resolution (HR) TEM. In Figure 2B, the uniformly dispersed composite particles showed a size of ~120 nm. The HRTEM image of 0.2% MoS_2_/Zn_0.5_Cd_0.5_S sample in Figure 2C demonstrated the construction of Zn_0.5_Cd_0.5_S solid solution again. Specifically, the smooth fringes of ~0.32 nm can be indexed to the (111) plane diffraction of Zn_0.5_Cd_0.5_S (Liu et al., 2011). Unfortunately, it is almost impossible to observe the lattice fringes of MoS_2_ owing to the low mass ratio of MoS_2_. To this end, the TEM image of 1% MoS_2_/Zn_0.5_Cd_0.5_S with a larger MoS_2_ content instead was analyzed by HRTEM, as shown in Figure 2D. In this case, the lattice spacing of 0.62 nm corresponding to the (002) planes of MoS_2_ can be clearly noticed (Zong et al., 2008), ensuring the successful coating of MoS_2_ on the surface of Zn_0.5_Cd_0.5_S. At the same time, it also testified the intimate tangency at atomic scale between MoS_2_ and Zn_0.5_Cd_0.5_S. In principle, this unique heterojunction structure should facilitate photogenerated charges separation and migration at the atomic scale (Zong et al., 2008; Akple et al., 2015; He et al., 2017).

**Figure 3:**
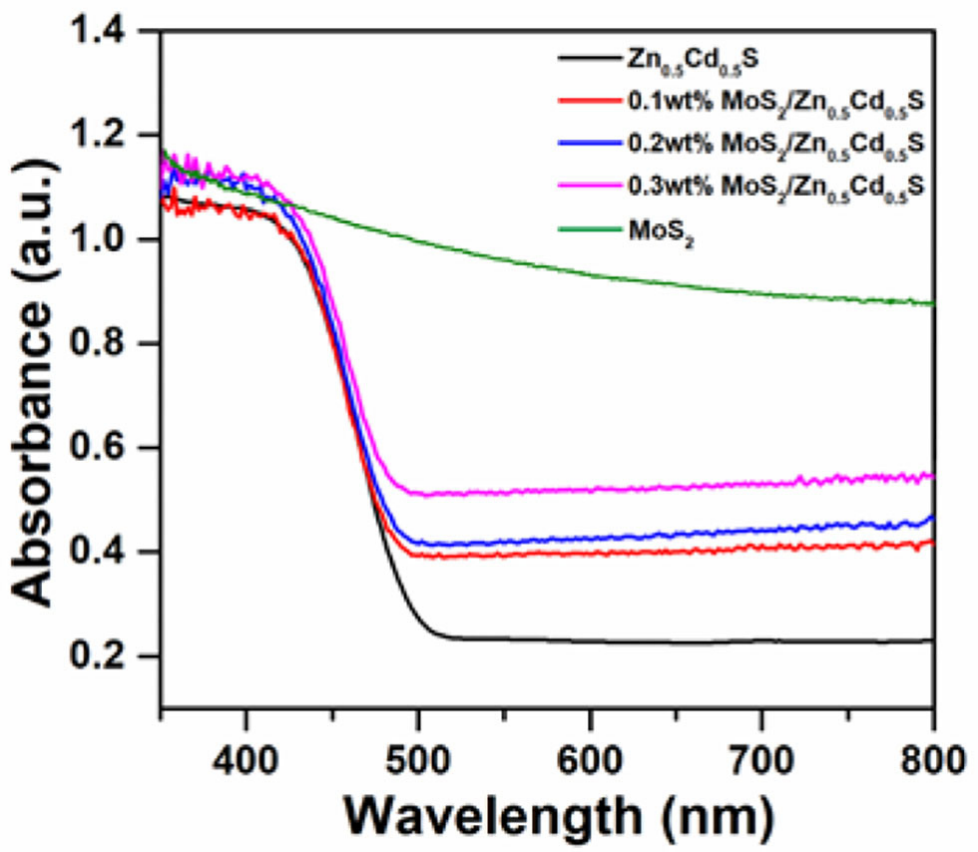
UV-vis spectra of pure MoS_2_ and MoS_2_/Zn_0.5_Cd_0.5_S with different molar fraction ratios.

To uncover the optical property of as-prepared composites, some crucial characterizations were then carried out. The UV-vis absorption spectra of MoS_2_/Zn_0.5_Cd_0.5_S samples are presented in Figure 3. The pure Zn_0.5_Cd_0.5_S nanocrystal exhibited an apparent absorption band edge at ~492 nm. The band gap of Zn_0.5_Cd_0.5_S thus can be estimated to be 2.52 eV, respectively. Furthermore, pristine MoS_2_ exhibited a strong light absorbance in the whole UV and visible region. The notion implies the metallic property of MoS_2_, foreboding a high potential to be cocatalyst. For the MoS_2_/Zn_0.5_Cd_0.5_S composites, they showed no distinct difference in absorption band. Nonetheless, the light absorption at longer wavelength between 520 and 800 nm was obviously increased. This enhancement was gradually amplified with the increased content of MoS_2_. All the phenomena above collectively confirm that the inherent band structure of Zn_0.5_Cd_0.5_S is well-preserved even after coupling MoS_2_, indicating that MoS_2_ was merely adhered on the surface of Zn_0.5_Cd_0.5_S tightly rather than Mo^4+^ incorporated into the Zn_0.5_Cd_0.5_S crystal lattice.

**Figure 4:**
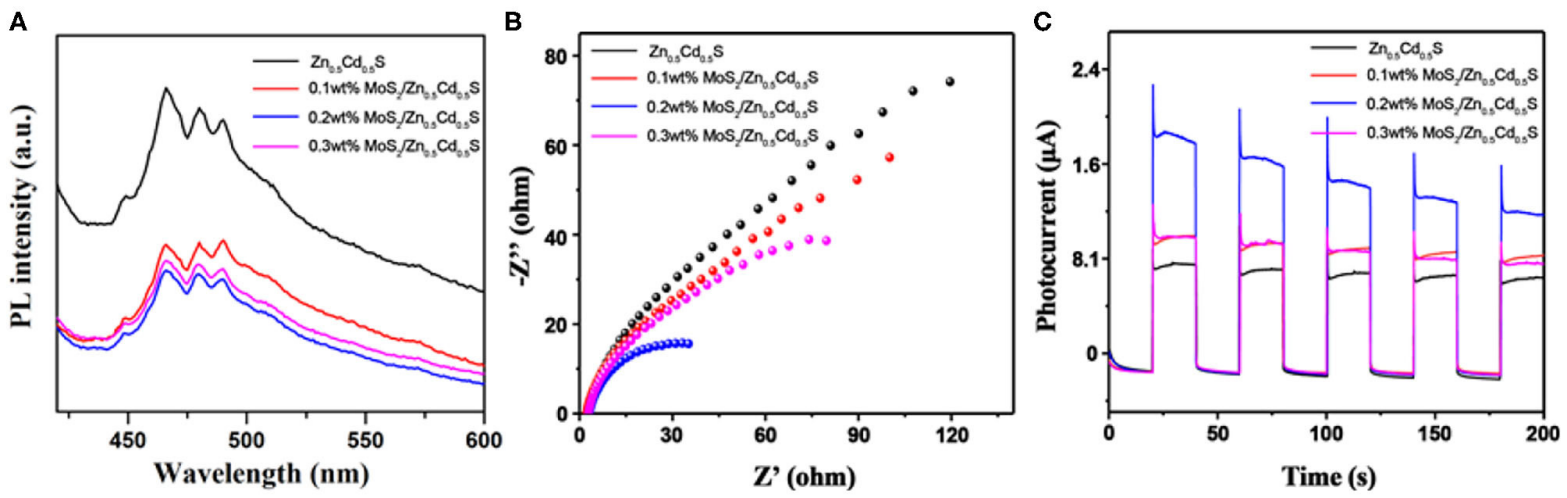
**(A)** PL spectra of different composite photocatalysts measured at an excitation wavelength of 325 nm. **(B)** The Nyquist plots of electrochemical impedance spectroscopy of various photocatalysts. **(C)** The corresponding transient photocurrent responses (I-t curves) of various photocatalysts under the Xe lamp irradiation.

Subsequently, the composite photocatalysts were investigated by PL. The spectra with an excitation wavelength of 325 nm are displayed in Figure 4. Pure Zn_0.5_Cd_0.5_S exhibited a strong intrinsic emission peak, indicating severe recombination of free carriers in Zn_0.5_Cd_0.5_S. As MoS_2_ was integrated with Zn_0.5_Cd_0.5_S, a similar spectrum trend with significantly quenched PL intensity of composites was observed, confirming the crucial function of MoS_2_ in accelerating the charge carriers transfer (Wang et al., 2019, 2020a,b). The optimized photogenerated carriers' dynamics also can be concluded from some other photoelectrochemical characterizations, including electrochemical impedance spectroscopy (EIS) and transient photocurrent response. As exhibited in Figure 4B, the smaller radius on the Nyquist plots of composites than that of pure Zn_0.5_Cd_0.5_S verifies the dramatically promoted electronic conductivity from Zn_0.5_Cd_0.5_S to MoS_2_ (Wang et al., 2019, 2020a,b). Meanwhile, the smallest semicircle of 0.2% MoS_2_/Zn_0.5_Cd_0.5_S certifies the content superiority. Figure 4C shows the transient photocurrent responses of samples in chopped illumination cycles. The enhanced photocurrent response of composite indicates the significant role of MoS_2_ in accelerating charge separation and improving surface reaction kinetics (Wang et al., 2019, 2020a,b). Notably, a small content of MoS_2_ (i.e., 0.1% MoS_2_/Zn_0.5_Cd_0.5_S) could induce insufficient active sites in the catalyst, whereas a large amount of MoS_2_ (i.e., 0.3% MoS_2_/Zn_0.5_Cd_0.5_S) would introduce new recombination centers. Both situations are unfavorable to depress the recombination rate of carriers and thus with limited ability to boost the redox reaction. As a result, 0.2% MoS_2_/Zn_0.5_Cd_0.5_S with the lowest PL signal, smallest EIS radius, and highest photocurrent could be expected to have a higher photocatalytic performance (Bi et al., 2016).

**Figure 5:**
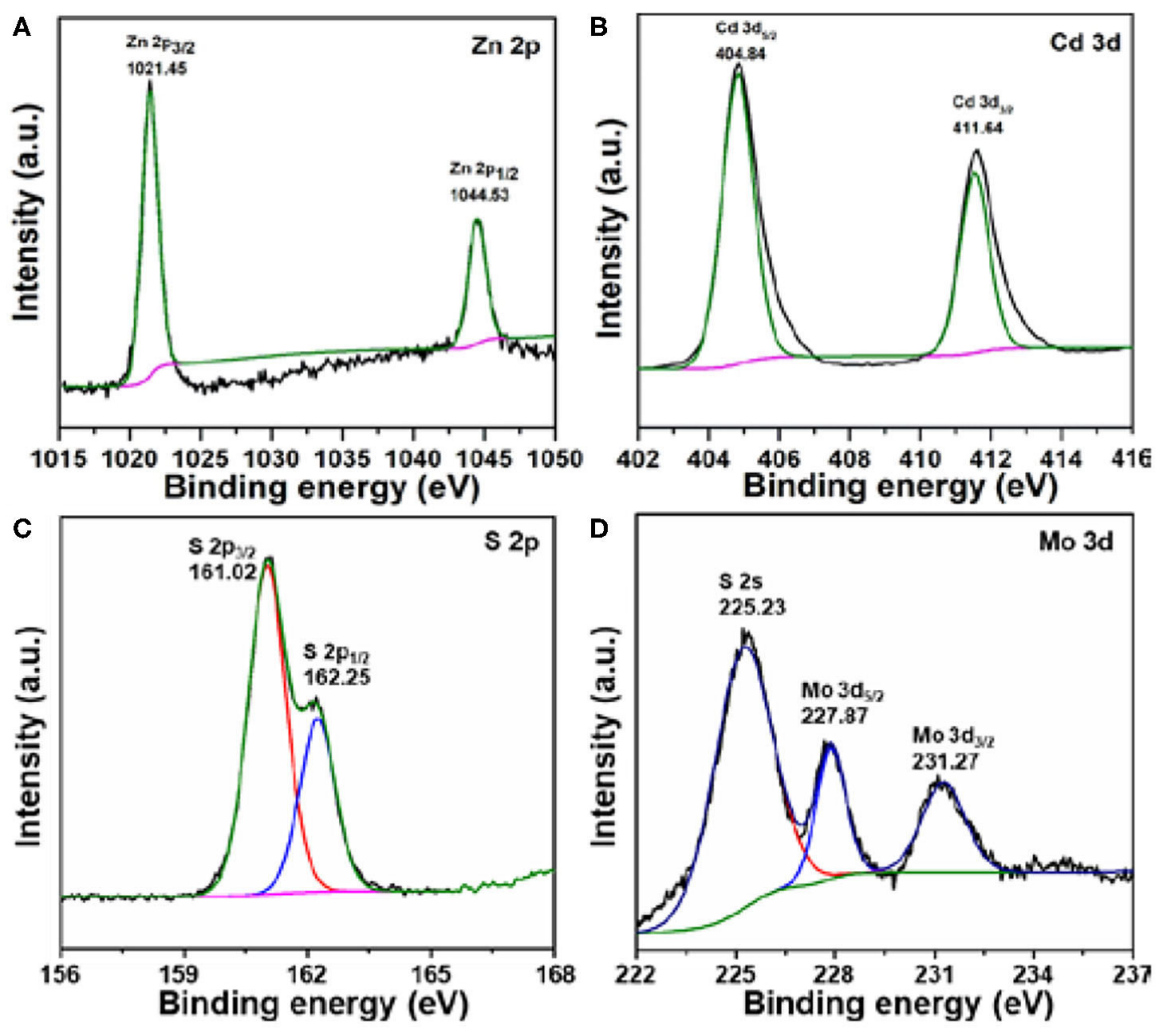
XPS spectra of **(A)** Zn 2p, **(B)** Cd 3d, **(C)** S 2p, and **(D)** Mo 3d in 0.2% MoS_2_/Zn_0.5_Cd_0.5_S.

We next sought to understand the surface chemical states by using XPS analysis. Taking 0.2% MoS_2_/Zn_0.5_Cd_0.5_S as an example, the XPS survey scans shown in Figure 5 revealed the existence of all the expected elements. The tiny peak intensity of Mo certified the low content in composite. The HR Zn 2p XPS spectra displayed two peaks at binding energies of 1,021.45 and 1,044.53 eV, which can be assigned to Zn 2p_3/2_ and Zn 2p_1/2_, respectively (Figure 5A; Qin et al., 2017). Meanwhile, the two well-defined peaks centered at 404.84 and 411.64 eV in the HR Cd 3d XPS spectra represented Cd 3d_5/2_ and Cd 3d_3/2_, respectively (Figure 5B). For sulfur, it was demonstrated in the chemical state of S^2−^ (Figure 5C). The above analysis provided further proofs on the formation of Zn_0.5_Cd_0.5_S solid solution. Furthermore, the peaks of Mo 3d appearing at binding energies of 227.87 and 231.27 eV belong to Mo 3d_5/2_ and Mo 3d_3/2_, respectively. It indicates that Mo^4+^ is the dominant chemical state (Figure 5D). More importantly, these peaks solidly indicate the 1T phase for MoS_2_ in 0.2% MoS_2_/Zn_0.5_Cd_0.5_S (2H at 229.0 and 232.3 eV) (Wang et al., 2013). Generally, MoS_2_ with 1T phase can function as a cocatalyst because of its metallic character (Bai et al., 2015). These results evidently confirm the successful synthesis of MoS_2_ cocatalyst (He et al., 2016; Zhang et al., 2018). Collectively, the XPS analysis further confirmed the coexistence of MoS_2_ and Zn_0.5_Cd_0.5_S solid solution in composites.

**Figure 6:**
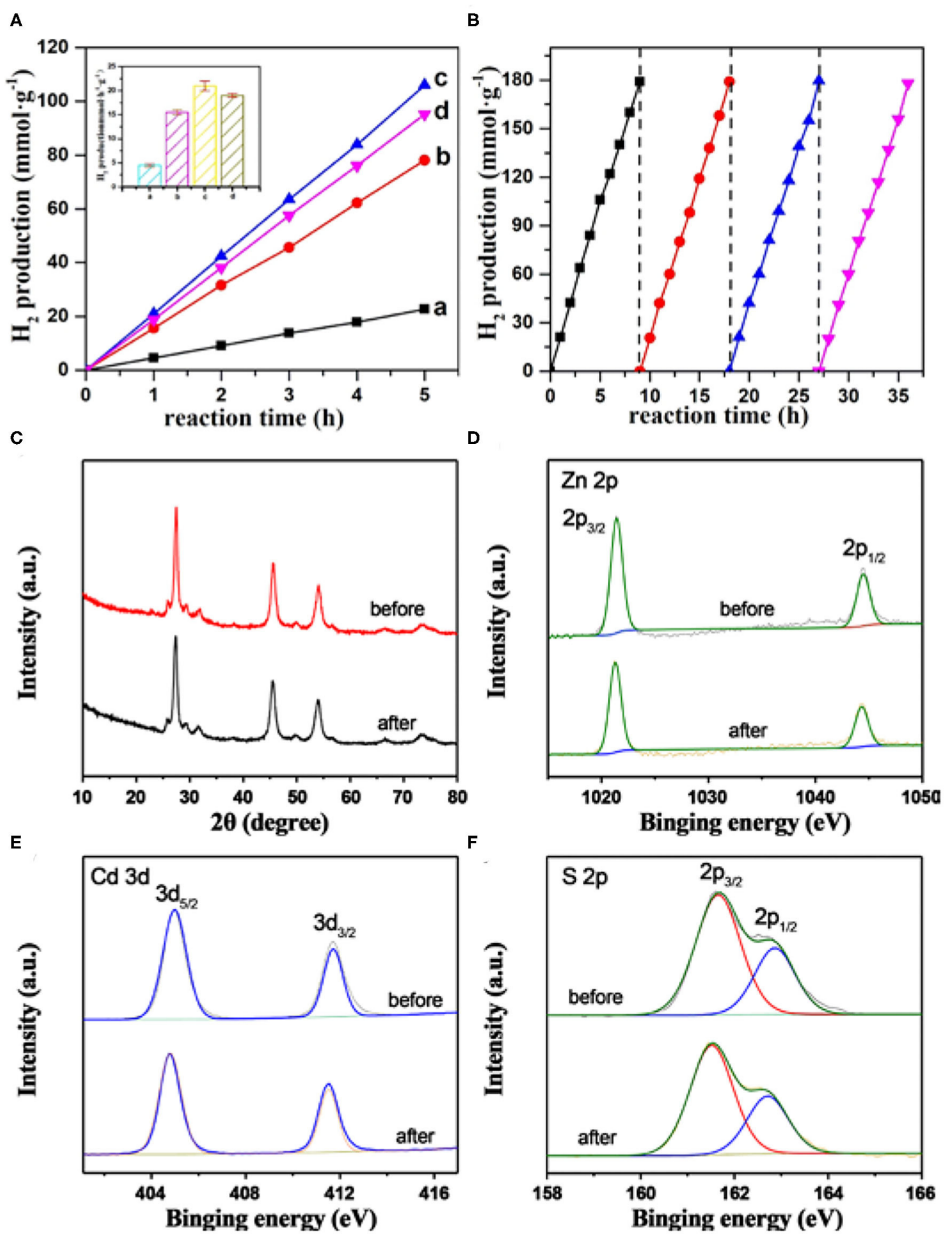
**(A)** Time-coursed photocatalytic H_2_ evolution activity of MoS_2_/Zn_0.5_Cd_0.5_S composites with various MoS_2_ content in aqueous solution containing 0.25M Na_2_S and 0.35 M Na_2_SO_3_. Herein, (a) Zn_0.5_Cd_0.5_S, (b) 0.1% MoS_2_/Zn_0.5_Cd_0.5_S, (c) 0.2% MoS_2_/Zn_0.5_Cd_0.5_S, (d) 0.3% MoS_2_/Zn_0.5_Cd_0.5_S. **(B)** Time courses of recycling tests toward photocatalytic H_2_ evolution over 0.2% MoS_2_/Zn_0.5_Cd_0.5_S composite photocatalyst. **(C)** XRD patterns and XPS spectra of **(D)** Zn 2p, **(E)** Cd 3d, **(F)** S 2p in 0.2% MoS_2_/Zn_0.5_Cd_0.5_S before and after 3-time recycling tests. The inset in **(A)** is the photocatalytic H_2_ evolution rate of MoS_2_/Zn_0.5_Cd_0.5_S composites with various MoS_2_ content.

The photocatalytic activity toward H_2_ production from water over the MoS_2_/Zn_0.5_Cd_0.5_S composite photocatalysts was then evaluated under visible-light irradiation (Figure 6A). No H_2_ evolution was detected without either illumination or photocatalyst, indicating the photocatalytic function of MoS_2_/Zn_0.5_Cd_0.5_S composite photocatalysts. As displayed in Figure 6A, the photocatalytic hydrogen production via water splitting increased linearly with time. Pure Zn_0.5_Cd_0.5_S showed a low photocatalytic activity due to the rapid recombination of electron-hole pairs. While the introduction of the MoS_2_ cocatalyst gave rise to a significant improvement in the photocatalytic H_2_ evolution, this phenomenon can be explained by the fact that MoS_2_ can effectively extract electron from Zn_0.5_Cd_0.5_S and act as the active site of photocatalytic H_2_ production. More importantly, an MoS_2_ content–dependent volcano-type trend for the activity was also acquired. With a low content of MoS_2_, although the charge transfer dynamics can be accelerated in some degree, the insufficient active sites restrict the extraction of electrons from Zn_0.5_Cd_0.5_S and the surface reaction rates. However, too much MoS_2_ introduction will impede optical absorption, shield the photoactive sites, and induce new recombination centers. Therefore, the hydrogen production rate was reduced in the two cases. With the optimal loading amount of cocatalyst, 0.2% MoS_2_/Zn_0.5_Cd_0.5_S showed the highest hydrogen production rate of 21 mmol · h^−1^ · g^−1^, deriving from the appropriate photoabsorption, charge transfer rate, and active sites. The activity is four times higher than that of Zn_0.5_Cd_0.5_S. Meanwhile, the AQY of 0.2% MoS_2_/Zn_0.5_Cd_0.5_S was also measured to be as high as 46.3% at 425 nm. The photostability of the composite photocatalysts was also evaluated by taking 0.2% MoS_2_/Zn_0.5_Cd_0.5_S as an example. As seen from Figure 6B, continuous hydrogen output with constant rate over 0.2% MoS_2_/Zn_0.5_Cd_0.5_S was clearly appreciable, even if this reaction proceeded for more than 30 h. The above result enunciates the desirable chemical stability of the as-prepared composite photocatalysts. Moreover, the XRD (Figure 6C) and XPS (Figures 6D–F) examination of the 0.2% MoS_2_/Zn_0.5_Cd_0.5_S after a three-time recycling test, without unveiling non-detectable alternations relative to fresh sample, provided further evidence for the sufficient protection of MoS_2_ to the primary Zn_0.5_Cd_0.5_S photocatalyst.

**Figure 7:**
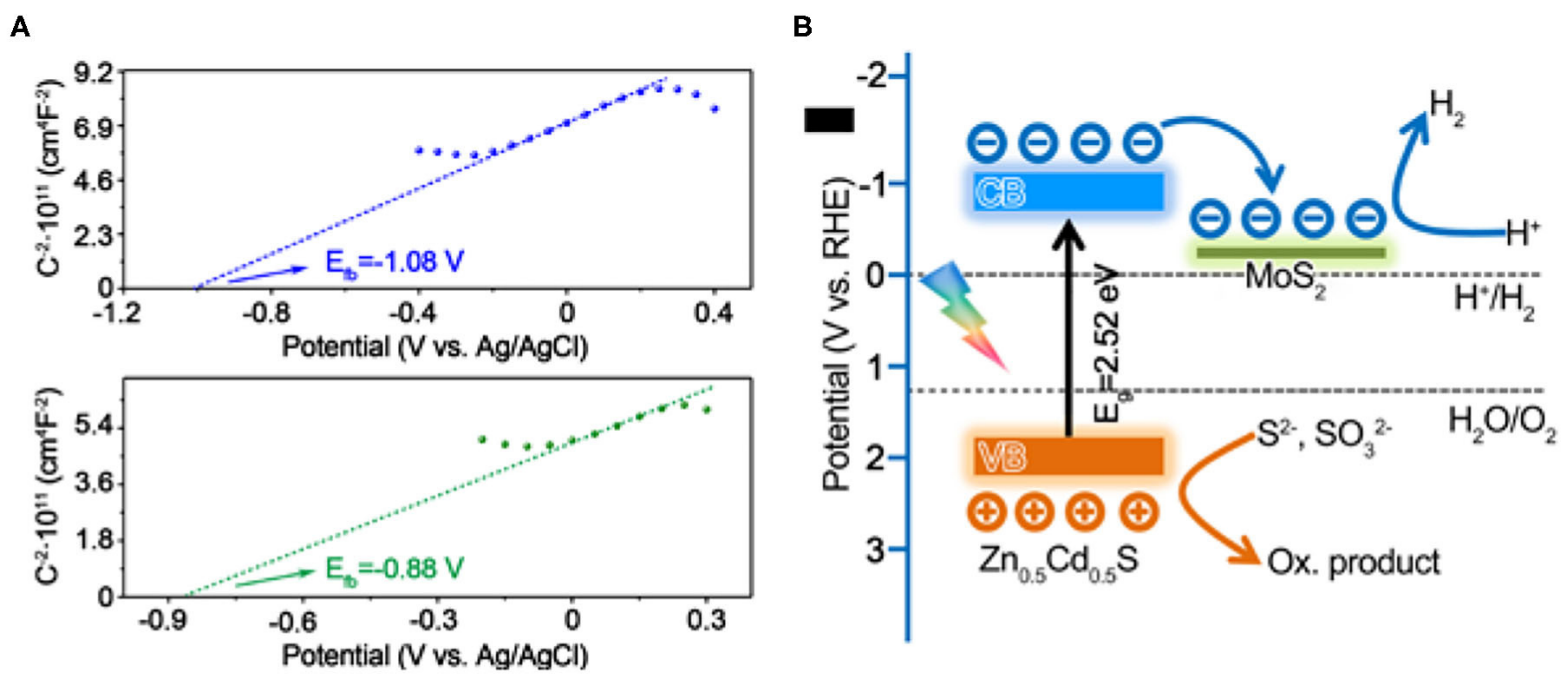
The Mott–Schottky plots for **(A)** Zn_0.5_Cd_0.5_S (upper part) and MoS_2_ (bottom part). **(B)** Proposed photocatalytic charge separation process over the band-structure controlled MoS_2_/Zn_0.5_Cd_0.5_S photocatalyst for efficiently sacrificially solar hydrogen production.

In order to acquire the photocatalytic mechanism of the composite, the band alignment should be determined first. The exact band level can be determined by an intercept method in MS plots together with the band gap derived from the UV-vis absorbance spectra. As exhibited in Figure 7A, the flat band potentials (E_fb_) of Zn_0.5_Cd_0.5_S and MoS_2_ were estimated to be −0.47 and −0.27 V (vs. RHE), respectively. Generally, the CB position is considered to be more negative by 0.2 eV than E_fb_ for semiconductor. Accordingly, the authentic CB for Zn_0.5_Cd_0.5_S is −0.67 V (vs. RHE). Combining the band gap of Zn_0.5_Cd_0.5_S that was determined to be 2.52 eV, the VB of Zn_0.5_Cd_0.5_S is calculated to be 1.85 V (vs. RHE), respectively, while for the metallic MoS_2_, the flat band potentials approximately equal its Fermi level (E_f_) (Ran et al., 2017). Based on the above analysis, the photocatalytic pathway over MoS_2_/Zn_0.5_Cd_0.5_S composite photocatalysts was illustrated in Figure 7B. From a thermodynamic viewpoint, both E_g_ and E_CB_ of Zn_0.5_Cd_0.5_S satisfy the precondition for photocatalytic H_2_ generation. Hence, this reaction process under assistant of Zn_0.5_Cd_0.5_S would take place very smoothly. Upon exposing to visible light, the electrons in VB of Zn_0.5_Cd_0.5_S will be excited to its CB to induce electron-hole pairs. Because of the thermodynamic driving force induced by the distance between low Fermi level of MoS_2_ and high CB of Zn_0.5_Cd_0.5_S, together with the small interface barriers that stemmed from strong interaction between MoS_2_ and Zn_0.5_Cd_0.5_S caused by one-pot hydrothermal treatment, the electrons on Zn_0.5_Cd_0.5_S will directionally migrate to MoS_2_, leading to a spatial separation of these charge carries (Chang et al., 2015; Yin et al., 2016). As a result, the protons will be effectively reduced to be H_2_ at active sites provided by MoS_2_, accompanied by transfer of holes to surface of Zn_0.5_Cd_0.5_S for consumption by the sacrificial agents, leading to the promotion of photocatalytic activity over these MoS_2_/Zn_0.5_Cd_0.5_S composites.

## Conclusions

In summary, a series of MoS_2_/Zn_0.5_Cd_0.5_S composite photocatalysts have been successfully constructed. The success relies upon a mild one-pot hydrothermal process for incorporating MoS_2_ on the surface of Zn_0.5_Cd_0.5_S solid solution in identical growth environment. All the composite photocatalysts showed significant enhancement in solar hydrogen evolution. The highest photoactivity was achieved over the 0.2% MoS_2_/Zn_0.5_Cd_0.5_S photocatalyst, with an H_2_ production rate of 21 mmol · h^−1^ · g^−1^ and an AQY of 46.3% at 425 nm, highlighting the intense promoting effect of MoS_2_ as a cocatalyst. Specifically, MoS_2_ can steer the electrons through the interface between these two components by strong interfacial interaction formed in a one-pot hydrothermal method. Meanwhile, abundant active sites provided by MoS_2_ also can facilitate the surface redox reaction. This work provides the feasibility to develop noble metal–free cocatalyst incorporating a photocatalyst system for efficient and stable H_2_ evolution.

## Data Availability Statement

All datasets presented in this study are included in the article/supplementary material.

## Author Contributions

XL and FX: conceptualization, methodology, formal analysis, writing—original draft, visualization, and data curation. NL: methodology, formal analysis, and data curation. XW: experimental assistant. HL: writing—review and editing, and project administration. JZ and BL: result discussion. ML: conceptualization, methodology, writing—review and editing, supervision, project administration, and funding acquisition. All authors contributed to the article and approved the submitted version.

## Conflict of Interest

The authors declare that the research was conducted in the absence of any commercial or financial relationships that could be construed as a potential conflict of interest.
